# The Cerebellum Is a Key Structure in the Neural Network for Mentalizing: An MRI Study in the Behavioral Variant of Frontotemporal Dementia

**DOI:** 10.3390/biomedicines10112901

**Published:** 2022-11-11

**Authors:** Giusy Olivito, Davide Quaranta, Libera Siciliano, Naike Caraglia, Alessia Caprara, Camillo Marra, Maria Leggio, Maria Caterina Silveri

**Affiliations:** 1 Department of Psychology, Sapienza University of Rome, 00185 Roma, Italy; 2 Ataxia Research Laboratory, IRCCS Santa Lucia Foundation, 00179 Rome, Italy; 3 Neurology Unit, Fondazione Policlinico Universitario “A. Gemelli” IRCCS, 00168 Rome, Italy; 4 Department of Neuroscience, Università Cattolica del Sacro Cuore, 00168 Rome, Italy; 5 Department of Psychology, Università Cattolica del Sacro Cuore, 20123 Milan, Italy; 6 Centre for the Medicine of Aging, Fondazione Policlinico Universitario “A. Gemelli” IRCCS, 00168 Rome, Italy

**Keywords:** functional connectivity, theory of mind, seed-based analysis, social cognition, voxel-based morphometry

## Abstract

The behavioural variant of frontotemporal dementia (bvFTD) is primarily characterized by deficits in social behaviour and theory of mind (ToM). Although a consensus has been reached on the roles of the cerebellum in social cognition and ToM, its specific contribution to social impairments of bvFTD has never been specifically investigated. The aim of this study was to assess cerebellar structural and functional changes in patients with bvFTD and their potential association with ToM deficits of patients. Therefore, 15 patients with bvFTD and 34 healthy subjects underwent an MRI examination. Voxel-based morphometry was used to assess cerebellar (GM) changes, and a seed-based analysis was performed to test cerebello-cerebral functional connectivity (FC). The performance of bvFTD patients in a ToM task was then correlated with FC patterns. Compared to healthy subjects, patients with bvFTD showed significant cerebellar GM loss specifically involving cerebellar Crus I-II. Additionally, FC changes FC were observed between the cerebellum and cerebral regions related to ToM. Interestingly, patterns of changes in cerebello-cerebral FC correlated with altered ToM performances explored using the “Reading the Mind with the Eyes” test (RMET) of patients. The present findings suggest that specific changes in cerebello-cerebral FC may underlie ToM alterations in patients with bvFTD.

## 1. Introduction

It is widely acknowledged that damage to the cerebellum is accompanied by motor symptoms and nonmotor manifestations that constitute the so-called cerebellar cognitive-affective syndrome [1]. Cerebellar cognitive-affective syndrome includes both cognitive deficits, involving executive, visuospatial, language, and attention domains [2,3,4,5], and affective behavioural manifestations characterized by emotional flattening or reduced emotional control [2] disinhibition and inappropriate behaviors. More recently, the cerebellar contribution has also been extended to social cognition [6], a broad group of cognitive operations involved in processing and interpreting socially relevant stimuli, including theory of mind (ToM) [7]. ToM, or the mentalizing process, is a fundamental aspect of social cognition (SC) and is crucial for social interactions; it refers to the ability to attribute mental states, such as emotion, intentions, and beliefs, to others to explain and predict their behaviors [8,9]. ToM functions mainly depend on a set of brain regions called the “mentalizing network”, including areas of the temporoparietal junction, medial prefrontal cortex [10,11], the precuneus [12], amygdala [13], insula [14,15], and middle temporal gyrus [16].

Interestingly, functional magnetic resonance imaging (fMRI) studies have reported the presence of functional connectivity (FC) between specific cerebellar regions, such as Crus I and II, and mentalizing cerebral regions [12,17,18]. This evidence suggests that the involvement of the cerebellum in ToM depends on its functional connectivity with the cerebrum [19].

Accordingly, ToM deficits have been described in patients with neurodegenerative diseases of the cerebellum [20,21,22]. On the other hand, cerebellar alterations have been reported in pathological conditions typically characterized by social and mentalizing dysfunctions, such as autism spectrum disorders [23].

Deficits in social cognition have been recognized as a hallmark of the behavioural variant of frontotemporal dementia (bvFTD) [7], representing one of the reliable markers for the differential diagnosis with respect to Alzheimer’s disease [24].

In patients with bvFTD, the cognitive manifestations may be not present, especially in the early stages, and are inconstant and often less severe than the behavioural disorder. Generally, no important memory disturbances are observed, while slight executive and language difficulties might arise. However, progressive changes in personality and social interaction typically precede other cognitive deficits [25]. In particular, the significant decline in recognition of mental states, as assessed by the Reading the Mind in the Eyes test (RMET, ref. [26]), may represent the best diagnostic predictor for bvFTD [27].

Beyond the distributed atrophy patterns in the cerebral cortex, the involvement of the cerebellum in dementia has been widely described [28]. In particular, in the cerebellum, distinct and circumscribed atrophy has been reported across different subtypes. This evidence suggests that the cerebellum may contribute to the cognitive and affective processes that are selectively compromised in different age-related neurodegenerative conditions [29]. In this framework, cerebellar circuits, which share extensive connections with the cerebral cortex, could be selectively targeted by major neurodegenerative diseases [29]. 

Although the most relevant neuropathological finding in patients with bvFTD is atrophy of the frontal and anterior temporal lobes [30], cerebral atrophy patterns also resemble the default mode network (DMN) [29], an intrinsic functional connectivity network including different mentalizing regions, i.e., the angular gyrus, precuneus, and posterior cingulate cortex, that are functionally related to the cerebellum [17,31]. Furthermore, a widespread pattern of cerebellar atrophy has been described in a recent study by Chen and colleagues [32], showing bilateral involvement of the cerebellar lobules, with the exception of lobules X and XI, and mainly affecting Crus I and II. These cerebellar regions have been consistently implicated in mentalizing functions and suggested to play a domain-specific role that is independent of the executive domain [12,20,33]. Despite several lines of evidence of altered social performance in patients with bvFTD [27,34,35], no study has investigated the relationship between structural and functional patterns in the cerebellum and ToM alterations in patients with bvFTD. The present study specifically addresses this issue by integrating structural and functional MRI with behavioural data. The aim is to clarify whether specific cerebellar atrophy patterns might affect functional interactions within core mentalizing cerebellar and cerebral regions, thus impairing ToM functioning in patients with bvFTD.

## 2. Materials and Methods

### 2.1. Participants

Fifteen patients (mean age/SD: 69.8/5.6 years; M/F = 11/4; mean educational level/SD: 11.1/4.8 years) fulfilling clinical the diagnostic criteria for bvFTD [36] were screened among subjects referring to the Neuropsychology Unit of the Catholic University of Rome for memory and behavioural disorders. According to Rascovsky and colleagues [36], patients with bvFTD presenting at least three of the following symptoms at onset were enrolled:

(1) Disinhibition (socially inappropriate behaviour, loss of decorum, and impulsiveness)
(2) Apathy or inertia (quantitative reduction in purposeful voluntary behaviours)
(3) Loss of empathy
(4) Repetitive behaviours, ritualisms, or stereotypes
(5) Hyperorality (oral exploration of objects, substantial changes in food preferences, binge eating, and increased consumption of tobacco or alcohol)
(6) Cognitive modifications (deficit of executive functions with at least partial preservation of episodic memory and visuospatial skills).

For each patient, the diagnosis was supported by structural MRI and functional neuroimaging (positron emission tomography, PET, or single-photon emission computerized tomography, SPECT) performed prior to inclusion in the study. Specifically, atrophy and hypometabolism/hypoperfusion in the frontal regions was displayed as also extending to the anterior temporal lobes.

Each patient was administered the Mini-Mental State Examination (MMSE) [37] to evaluate global cognitive decline. Furthermore, an extensive neuropsychological battery was used to assess specific cognitive modifications (see the Supplementary Material for detailed descriptions).

Additionally, the Frontal Behavioural Inventory scale (FBI; ref. [38]) and the clinical dementia rating scale (CDR; ref. [39]) were used to assess behavioural features and the overall severity of dementia, respectively.

Exclusion criteria were the absence of an informed caregiver, unavailability of a neuroradiological examination, and/or the assumption of psychotropic drugs taken within two months prior to the clinical assessment.

Two different control groups with no history of neurological or psychiatric illnesses were enrolled, one for the MRI examinations and one for ToM assessment. A control group of 34 healthy subjects for the MRI examinations (HS-MRI) (mean age/SD: 69.1/6.6 years; M/F: 17/17; mean educational level/SD: 12.9/6.7 years) was enrolled based on retrospective MRI data collected from healthy participants over the last ten years at the Neuroimaging Laboratory of Santa Lucia Foundation. The statistical analysis showed no significant differences between the bvFTD and HS-MRI groups in age (T: 0.36342, *p* = 0.71), educational level (T: 1.42659, *p* = 0.16), and sex distribution (Chi2 = 1.4592, *p* = 0.22). Additionally, a group of 27 healthy subjects (HS-ToM) (mean age/SD: 67.2/5.9 years; M/F = 15/12 mean educational level/SD: 13.5/3.4 years) was enrolled for the assessment of ToM. The statistical analysis showed no significant differences between the bvFTD and HS-ToM groups in age (T: 1.39888, *p* = 1.000), educational level (T: −1.97166, *p* = 0.055), and sex distribution (Chi2 = 1.2923; *p* = 0.255623).

The sociodemographic, clinical, neuropsychological, and behavioural characteristics of the bvFTD group and control groups are reported in Table 1.

This research study was approved by the Ethics Committee of Santa Lucia Foundation and Policlinico Universitario A. Gemelli, according to the principles expressed in the Declaration of Helsinki. Written informed consent was obtained from each subject.

### 2.2. MRI Acquisition Protocol

All patients with bvFTD (n = 15) and the HS-MRI group (n = 34) underwent an MRI examination with a 3 T instrument (Philips, Achieva) that included the following acquisition sequences: (1) dual-echo turbo spin echo [TSE] (TR = 6190 ms, TE = 12/109 ms); (2) fast-FLAIR (TR = 8170 ms, 204 TE = 96 ms, and TI = 2100 ms); (3) T1-weighted 3D high-resolution scan 3D modified driven equilibrium Fourier transform [31] (TR = 1338 ms, TE = 2.4 ms, matrix = 256 × 224 × 176, in-plane FOV = 250 × 250 mm^2^, and slice thickness = 1 mm); and (4) T2*-weighted echo-planar imaging (EPI) sensitized to blood oxygenation level–dependent imaging contrast (TR, 2080 ms, TE 30 ms, 32 axial slices parallel to AC-PC line, matrix = 64 × 64, pixel size = 3 × 3 mm^2^, slice thickness = 2.5 mm, and flip angle = 70°) for resting-state fMRI. Blood oxygenation level-dependent echo planar images were collected during rest for 7 min and 20 s, resulting in a total of 220 volumes. During this acquisition, subjects were instructed to keep their eyes closed, not to think of anything in particular, and not to fall asleep. The TSE scans of patients with bvFTD that were acquired as part of this research study were reviewed by an expert neuroradiologist to characterise the brain anatomy. For the HS group, conventional MRI scans were inspected to exclude any pathological conditions according to the inclusion criteria.

### 2.3. Image Processing and Data Analysis

#### 2.3.1. Voxel-Based Morphometry

Voxel-based morphometry (VBM) was used to identify differences in regional cerebellar volume between the bvFTD and HS-MRI groups. The cerebellum was preprocessed individually using the Spatially Unbiased Infratentorial Template (SUIT) toolbox [49] implemented in statistical parametric mapping (Wellcome Department of Imaging Neuroscience; SPM-8 (http://www.fil.ion.ucl.ac.uk/spm/), accessed on 1 September 2022). The procedure was the same used in previous studies [5,20]. A voxelwise two-sample t test was used to assess between-group differences in regional GM cerebellar volumes. The cerebellum was entered as an explicit inclusion mask. The results were considered significant at *p* values < 0.05 after FWE cluster-level correction (clusters formed with *p* < 0.001 at an uncorrected level). Additionally, the MDEFT of each participant was also segmented in SPM to estimate the total grey matter (GM) volume in the bvFTD and control groups.

#### 2.3.2. Resting-State fMRI Data Preprocessing

SPM8 (http://www.fil.ion.ucl.ac.uk/spm/, accessed on 1 September 2022) was used to preprocess fMRI data and in-house software was implemented in MATLAB (The Mathworks Inc., Natick, MA, USA). For each subject, T1 equilibration effects were ensured by discarding the first four volumes of the fMRI series. For the detailed preprocessing steps, see Olivito and colleagues [50].

### 2.4. Definition of Regions of Interest (ROIs) and Seed-Based Analyses

**Figure 1:**
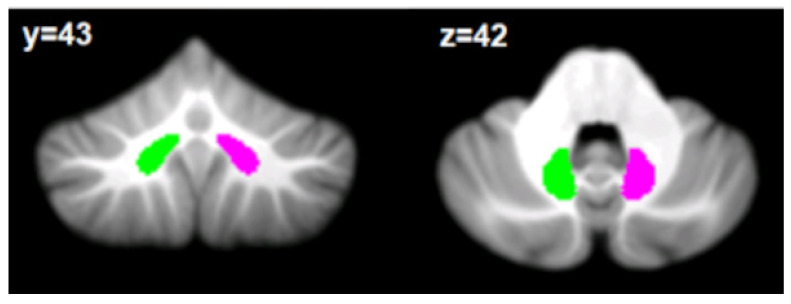
Seed regions in the cerebellar dentate nucleus. Coronal (y) and axial (z) view of the generated left (green) and right (violet) dentate nucleus superimposed to the spatially unbiased atlas template of the cerebellum and brainstem (SUIT, [49]).

Two different seed-based analyses were performed. Based on the VBM results, the most affected cerebellar regions were identified and used as regions of interest (ROIs) in the first seed-based analysis. According to the evidence that the cerebellar dentate nucleus (DN) represents one of the major cerebellar output channels [51], the left and right DN masks were separately used as ROIs for a second seed-based analysis (Figure 1).

According to the SUIT atlas template of the cerebellum [49], the regions with significantly reduced GM volume and both DNs were extracted by using the FSL command line from the fmrib software library (FSL, www.fmrib.ox.ac.uk/fsl/, accessed on 1 September 2022) and resliced into EPI standard space.

For every participant, the mean time course of the voxels within the ROIs was calculated and used as a regressor in a first-level SPM analysis, thus extracting the voxels from the whole brain and showing a significant correlation. At the second level, a two-sample t test was used to explore differences in connectivity in the identified ROIs between the bvFTD and HS-MRI groups. The total brain GM volume was entered into the analysis as a covariate of no interest to remove the confounding effect of the global atrophy pattern in patients with bvFTD. The cerebellum was excluded from the analysis by entering an implicit inclusion mask that included only the cerebral cortex. The results were considered significant at *p* values < 0.05 after FWE cluster-level correction (clusters formed with *p* < 0.001 at the uncorrected level).

### 2.5. ToM Assessment and Analysis

ToM was investigated using the RMET [26,52], which, as previously reported, represents one of the most sensitive tests to assess social cognitive impairment in patients with bvFTD [27]. This test evaluates the first (automatic) stage of mentalizing and specifically assesses the ability to attribute the relevant mental state (feelings and thoughts) to others when the mental state is not inferable from the stimuli. The participants are asked to match the mental state of persons shown in 36 photos of their eye regions by choosing, from a list of words, the one that best describes actor’s mental state. 

A nonparametric Mann-Whitney U test for independent samples was used to detect differences in the RMET accuracy row score between patients with bvFTD and HS-ToM. Statistical analyses were performed using the SPSS statistics package (version 25).

### 2.6. Behavioural Correlations with Functional Connectivity

Based on RS-fMRI data, the mean cerebello-cerebral FC values from clusters that were significantly altered in patients with bvFTD were extracted. Correlations between RMET accuracy scores and FC values were determined by calculating the Spearman correlation coefficient using the SPSS statistics package.

## 3. Results

### 3.1. Voxel-Based Morphometry

**Figure 2:**
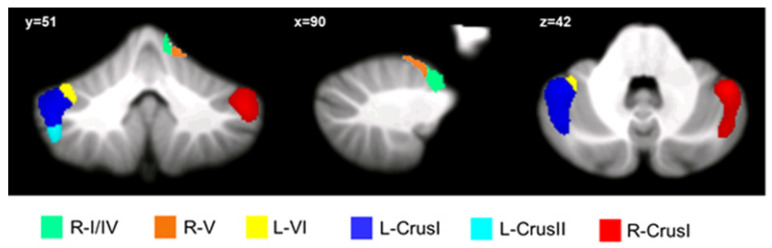
Between-group voxel-based comparison of cerebellar GM density. Cerebellar regions showing patterns of significantly reduced GM in bvFTD are reported and superimposed on the Spatially Unbiased Infratentorial Template (SUIT) [49] in coronal (y), sagittal (x), and axial (z) slices. The results are significant at *p*-values < 0.05 after I cluster-level correction. Images are shown in neurological convention. R: right, L: left.

The results showed the presence of structural alterations in patients with bvFTD compared to the HS-MRI group at the level of the cerebellar hemispheres. Specifically, patients with bvFTD showed significant clusters of reduced GM density involving right lobules I–IV and V, left lobule VI, right and left Crus I, and left Crus II (Figure 2). Detailed statistics and peak voxels showing the greatest significant differences in a cluster are reported in Table 2.

### 3.2. Seed-Based FC Results

**Figure 3:**
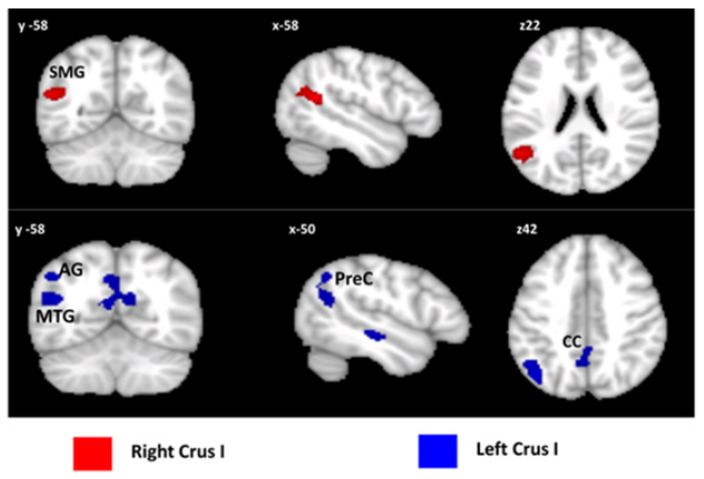
Patterns of cerebellar FC with the cerebral cortex. Seed-to voxel patterns of increased FC in bvFTD patients for right (in red) and left (in blue) Crus I. Coronal (y), sagittal (x), and axial slices (z) in the Montreal Neurological Institute space. Clusters of increased FC in the cerebral cortex were considered significant after correction for multiple comparisons (FWE-corrected *p* < 0.05). Images are shown in neurological convention. SMG: supramarginal gyrus; AG: angular gyrus; MTG: middle temporal gyrus; Prec: precuneus; CC: cingulate cortex.

No subject was excluded due to motion artefacts. According to the voxelwise analysis, the left and right cerebellar Crus I and the left Crus II were the most affected regions in patients with bvFTD compared to the HS-MRI group and were used as ROIs for the seed-based analysis. Compared to controls, patients with bvFTD showed a pattern of increased FC between the left and right Crus I and cerebral cortex regions, while no pattern of decreased FC was detected. Specifically, a single cluster of significantly increased FC was observed between the right Crus I and the left angular gyrus, with extension to the left supramarginal gyrus. Additionally, different cluster-level peaks displaying increased FC were observed, involving the left Crus I and the right precuneus, the left parahippocampal gyrus, the left lateral occipital cortex, the left angular gyrus, and the left middle temporal gyrus. No patterns of altered FC were detected between the left Crus II and cerebral cortex (Figure 3).

**Figure 4:**
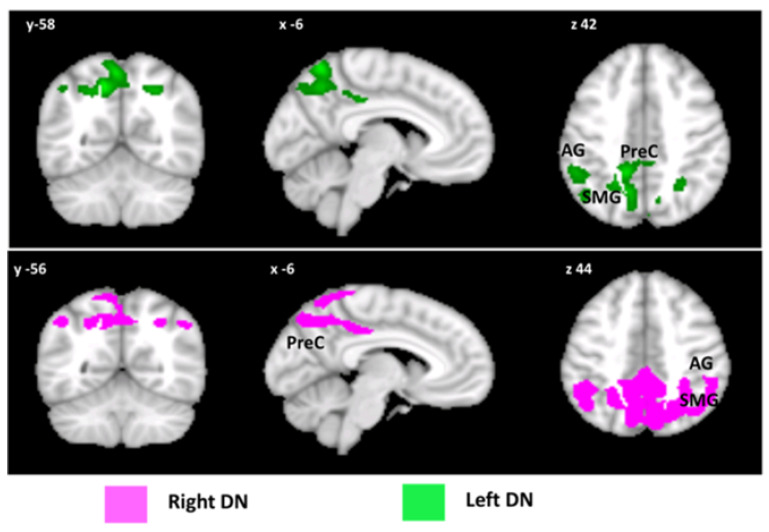
Patterns of DN FC with the cerebral cortex. Seed-to voxel patterns of increased right (violet) and left (green) DN FC with the cerebral cortex. Coronal (y), sagittal (x), and axial slices (z) in the Montreal Neurological Institute space. Clusters of increased FC in the cerebral cortex were considered significant after correction for multiple comparisons (FWE-corrected *p* < 0.05). Images are shown in neurological convention. AG: angular gyrus; SMG: supramarginal gyrus; Prec: precuneus.

Finally, the analyses of DN FC also showed patterns of significantly altered FC. In particular, increased FC was observed between the left DN and different clusters in the cerebral cortex involving the left precuneus, the left lateral occipital cortex and the left supramarginal gyrus. Additionally, increased FC was observed between the right DN and clusters involving the bilateral precuneus, the left and right lateral occipital cortex, the left supramarginal gyrus and the left angular gyrus. No patterns of decreased FC were detected (Figure 4). Detailed statistics of seed-based analyses are reported in Table 3.

### 3.3. ToM Assessment and Correlation with Cerebello-Cerebral FC

According to post-hoc power analyses using the G-power software and estimating effect size from data on RMET performances (large effect size, d = <0.8), 99% of power (alfa: 0.05) is expected by using the non-parametric Mann–Whitney U test with a population of 15 patients and 27 healthy subjects. 

In the RMET, significantly lower accuracy scores (MWU: 20.500; Z: −4.78; *p*: 0.000) were recorded for patients with bvFTD (mean/SD: 14.8/5.6) than the HS-ToM group (mean/SD: 26.2/4.2).

**Figure 5:**
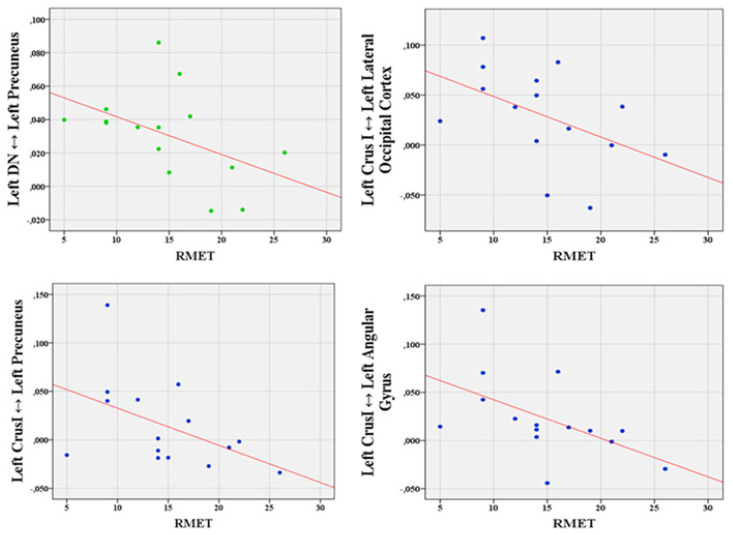
Scatterplots of correlations. Data scatterplots of significant correlations between RMET accuracy scores and cerebello-cerebral overconnectivity patterns. Correlations between RMET and left DN FC alterations are shown in the top panel in green; Correlations between RMET and left Crus I FC are shown in the bottom panel in blue. RMET: Reading the Mind in the eyes test [26]. DN: Dentate nucleus.

Interestingly, Spearman’s correlation analysis revealed significant correlations between impaired ToM performance and increased cerebello-cerebral FC in patients with bvFTD. Specifically, the RMET accuracy scores were negatively correlated with pairwise increased cerebello-cerebral FC between the left Crus I and left angular gyrus (r = −0.651, *p* = 0.004), left Crus I and left lateral occipital cortex (r = −0.532, *p* = 0.021), left Crus I and left precuneus (r = −0.446, *p* = 0.048), and left DN and the left precuneus (r = −0.543, *p* = 0.018). The scatterplots of the significant correlations are shown in Figure 5.

## 4. Discussion

The present RS-fMRI study provides the first evidence of altered FC within cerebellar and cerebral networks related to mentalizing in patients with bvFTD. The importance of the cerebellum in social/mentalizing functions has been recently acknowledged [12,53,54]. Several fMRI studies have documented the activation of specific cerebellar areas, such as Crus I and Crus II, during classic mirror tasks [53], and belong to the salience network (SN) and to the default mode network (DMN) [17,18]. Consistently, ToM deficits and FC alterations within mentalizing cerebello-cerebral networks have been described in patients affected by cerebellar pathologies [20,22]). Furthermore, a relationship between structural cerebellar changes and social dysfunction has been described in subjects with autism spectrum disorders [23].

The accumulating evidence indicating that the cerebellum is recruited in social cognition processing has led to the development of diverse hypotheses that attempt to explain this involvement. The activation likelihood estimation (ALE) meta-analysis conducted By Van Overwalle and colleagues [53] found an extensive overlap between areas of the cerebellum consistently involved in social cognitive processes and the areas involved in sensorimotor (during mirror and self-judgements tasks) and executive functioning [53]. According to this evidence, the authors proposed that the cerebellum is critically implicated in social cognition, especially when more complex and abstract social cognitive processes are required, thus suggesting that specific cerebellar zones have domain-general executive and semantic support [53]. Later, a multistudy connectivity analysis showed that cerebellar activity during social processes reflects a domain-specific mentalizing function that is independent of the executive domain and strongly connected with a corresponding mentalizing network in the cerebrum [12]. In the context of mentalizing functions, the DMN is of particular interest [55] since it includes a set of cerebral regions (i.e., the temporoparietal junction and precuneus) that are particularly relevant for the social understanding of others [56]. The functional segregation of the cerebellum has been observed in distinct resting-state fMRI studies [17,18,57], showing that the posterior cerebellar Crus I and II are functionally coupled to default mode regions specifically related to social mentalizing, while anterior Crus I is functionally associated with the cerebral frontoparietal network that is specifically related to executive functioning [58]. The results of the present study are highly consistent with these observations. In particular, cerebellar regions encompassing the left and right posterior Crus I, among others, were significantly affected in patients with bvFTD, as evidenced by reduced cerebellar GM.

Furthermore, we detected a pattern of functional overconnectivity between these cerebellar regions and mentalizing brain areas. Interestingly, a similar pattern of increased FC was observed between the DN and the mentalizing brain regions. The cerebellum is known to modulate cerebral cortical activity via cerebello-thalamo-cortical (CTC) circuits [59] and to selectively contribute to distinct functional networks that are clearly related to higher-level functions beyond motor control [18,60]. The cortical cerebellar inhibitory outputs converge onto the dentate nucleus (DN), which, in turn, sends excitatory neural fibres to the thalamus and the cerebral cortex via the superior cerebellar peduncles, thus completing the CTC circuit [59]. Thus, the DN represents the major cerebellar output channel participating in CTC circuits through the inhibitory modulation of the cerebellar cortex. Therefore, we hypothesised that cerebellar damage in the posterior Crus I alters the inhibitory modulation that is normally exerted by the cerebellar cortex on the DN, which subsequently increases its excitatory outputs to the connected mentalizing brain regions. Consequently, we observed a pattern of cerebello-cerebral overconnectivity that is the pathological manifestation of an altered cerebello-cerebral functional interaction that might alter ToM performances of patients with bvFTD. Consistent with this hypothesis, the functional overconnectivity between mentalizing cerebellar and cerebral regions also correlates with low RMET accuracy scores of patients with bvFTD, indicating that the greater the increase in the FC, the lower the ToM performances of patients.

Previous research has already shown alterations in social cognition in patients with bvFTD [27,34,35]. In particular, a recent meta-analysis by Henry and colleagues [61] confirmed the central role of ToM by showing significantly higher and domain-specific impairments in ToM (and emotion recognition) in patients with bvFTD compared with control subjects and patients with Alzheimer’s disease. Interestingly, several studies have shown that ToM is a good diagnostic predictor for bvFTD [27,62,63,64]. Although major impairments in both social cognition and executive functions in patients with bvFTD have been suggested in the literature [64], ToM tests, with particular reference to the RMET, seem to be better and more disease-specific predictors for bvFTD than executive function tests [27].

Overall, very slight executive deficits were observed in our bvFTD cohort, and no FC alterations were shown to affect cerebello-fronto-parietal networks. Interestingly, the pattern of correlations we found is consistent with the evidence that the temporoparietal junction encompassing the angular gyrus is a core region of the neural substrate for ToM, extending to several cerebral regions that also include the precuneus to constitute an extended ToM neural network [65,66].

Previous studies have shown that the cerebellum could be selectively targeted by neurodegenerative disorders, thus suggesting a network selective vulnerability of the cerebellum to different dementia subtypes [29].

In the context of frontotemporal dementia, cerebellar GM atrophy has been showed in all frontotemporal dementia subtypes, with particularly pronounced changes in patients with bvFTD, thus confirming that cerebellar changes are syndrome-specific and might partially reflect the disruption of specific cerebellar–cerebral connections [32]. This finding is also consistent with a previous meta-analysis study showing that cerebellar changes are largely disease-specific and correspond to cortical or subcortical changes in patients with neurodegenerative conditions, also including bvFTD [67]. Thus, the concomitant degeneration of interconnected infra- and supra-tentorial regions indicates connectivity-mediated propagation mechanisms and suggests that, although under-investigated, cerebellar degeneration is an important facet of bvFTD [68].

With respect to our results, an important issue that deserves to be discussed is the pattern of altered FC observed between cerebellar regions and cerebral areas in the ipsilateral hemisphere. Indeed, this result may be somewhat unexpected because the majority of cerebello-cerebral connections are contralateral [69,70]. However, lesional studies in rodents have documented bilateral cerebellar effects on the cerebral cortex, showing abnormal activity in the ipsilesional sensorimotor cortex [71] and ipsilateral connections between the cerebellum and cerebral cortex have also been identified [72,73,74]. Furthermore, functional connectivity might be partially independent of the underlying structural connections [75], since it refers to the functionally integrated relationship between spatially separated brain regions [76].

In conclusion, some concerns deserve discussion. Although the present results represent the first preliminary evidence of the cerebellar contribution to ToM deficits in patients with bvFTD, the mechanism by which the cerebellum specifically intervenes in mentalizing processes remains to be clarified. According to the cerebellar sequencing hypothesis [77], the cerebellum may contribute to ToM functions by detecting and memorizing patterns, constructing internal models of the perceived patterns, and comparing activity patterns to compute discrepancies in the same way that it acts in motor and other cognitive domains [78].

Importantly, this predictive and sequential coding is a central component of socioemotional processing [79] since the creation of a mental model of a mental state but also the ability to simulate how it might influence others’ behaviours are crucial to understand and inference others’ mental state. However, this issue is beyond the scope of the present study and was not specifically addressed. Another limitation might be related to the fact that only more automatic and basic components of ToM, as measured using the RMET, were investigated. Furthermore, the small sample size of patients with bvFTD might limit the significance and reproducibility of our findings. Nevertheless, the consistency between the present behavioural and MRI results and previous research supports the importance of our conclusions. However, future research should focus on investigating, in a larger bvFTD sample, both automatic and more complex ToM abilities, also including the possible dissociation between affective and cognitive components. This will allow us to better characterize the ToM profile of bvFTD patients as well as further elucidate whether structural/functional alterations of cerebellum and its networks are selectively linked to the impairment of specific ToM components. Overall, the present results shed further light on the importance of the cerebellum in cognitive and emotional processes, thus suggesting the importance of considering the cerebellum in clinical settings with bvFTD patients and in developing novel therapeutic programs. 

## 5. Conclusions

Although preliminary, the present study provides the first evidence of the cerebellar contribution to ToM deficits in patients with bvFTD. Although the cerebellum is rarely considered in the clinical diagnosis of many neurodegenerative conditions, this finding provides new insights into the mechanisms mediating bvFTD symptomatology, thus showing a link between social behavioural difficulties and cerebellar damage. Overall, the present results suggest that the ToM impairment in patients with bvFTD, which was previously exclusively attributed to supratentorial regions, may in part arise from cerebellar alterations.

## Figures and Tables

**Table 1:** Sociodemographic, clinical, neuropsychological, and behavioral features of the bvFTD sample.

		bvFTD		HS-MRI		HDS-ToM	
**Sociodemographical and Clinical Variables**		**M**	**SD**	**M**	**SD**	**M**	**SD**
Age (months)		69.8	5.63	69.1	16.6	67.2	5.9
Education (years)		11.1	4.82	12.9	6.7	13.5	3.4
Sex (M/F)		11/4 (73%/27%)		17/17(50%/50%)		15/12 (55%44%)	
Illness duration (months)		34.1	16.06				
Clinical Dementia Rating scale (CDR) [39]		1.1	0.56				
**Neuropsychological assessment**	**Cut-off**	**M**	**SD**				
Mini Mental State Examination [37]	>23.80	24.9	4.67				
RAVLT: immediate recall * [40]	>28.53	26.8	8.85				
RAVLT: delayed recall * [40]	>4.69	3.3	2.31				
RAVLT: recognition accuracy * [40]	>0.88	0.7	0.15				
Rey-Osterrieth figure copy * [41]	>28.87	23.6	9.48				
Rey-Osterrieth figure recall * [41]	>9.46	6.9	4.39				
Digit span forward [42]	>4.26	5.1	1.13				
Digit span backward [42]	>2.65	3.1	0.99				
Corsi’s test forward [42]	>3.46	3.9	1.51				
Corsi’s test backward [42]	>3.08	3.3	1.28				
Raven’s Progressive Colored Matrices [43]	>18.96	18.9	8.47				
Copy of figures [40]	>7.18	9.1	3.05				
Copy of figures with landmarks * [40]	>61.85	56.3	24.35				
Phonological Verbal Fluency [44]	>17.35	19.1	7.66				
Semantic Verbal Fluency [44]	>9.28	10.5	6.48				
MFTC accuracy [45]	>0.869	0.8	0.16				
MFTC false alarms [45]	<2.77	2.2	5.09				
MFTC time of execution [45]	<135.73	78.3	47.87				
Stroop’s test: interference time * [46]	<36.92	67.7	54.91				
Stroop’s test: interference errors [46]	<4.24	3.5	3.29				
MWCST: categories [47]	>2	3.0	1.93				
MWCST: perseverative errors * [47]	<6.41	9.6	11.47				
Trail Making part A * [48]	<93	99.5	44.53				
Trail Making part B * [48]	<282	302.0	138.19				
Trail Making B-A * [48]	<186	202.5	100.54				
**Behavioral assessment**							
FBI: Apathy [38]		18.3	5.96				
FBI: Disinhibition [38]		11.5	6.68				
FBI: Total Score * [38]	<28.6	29.8	11.06				

* Pathological scores. RAVLT: Rey’s Auditory Verbal Learning Test; MFTC: Multiple Features Targets Cancellation; MWCST: Modified Wisconsin Card Sorting Test; FBI: Frontal Behavioral Inventory. M: Mean; SD: Standard Deviation (See supplementary materials for detailed description of neuropsychological assessment).

**Table 2:** Detailed statistics of voxel wise comparisons of cerebellar GM density (bvFTD < HS-MRI).

Regions	Size	Side	MNI Coordinates (mm)			Peak Z-Scores
			x	y	z	
Lobule I–IV	3568	R	9	−35	−13	5.25
Lobule V		R	23	−31	−22	4.78
			14	−45	−8	4.71
Crus I	4455	R	46	−47	−36	4.07
		R	42	−61	−21	3.87
		R	42	−62	−29	3.81
Lobule VI	4770	L	−40	−46	−30	4.00
Crus I		L	−44	−48	−38	3.86
		L	−44	−58	−36	3.62

Results are significant at *p* < 0.05 after FWE correction. MNI coordinates (x, y, and z) in the Montreal Neurological Institute space.

**Table 3:** Detailed statistics of voxel-wise comparison of cerebello-cerebral FC.

Cerebellar Regions	Cerebral Regions	Size (NoV)	Side	MNI Coordinates (mm)			Peak Z-Scores
				x	y	z	
**R-CrusI**	Angular gyrus	279	L	−48	−52	−18	4.45
			L	−54	−58	22	4.13
**L-Crus I**	Parahippocampal gyrus	267	L	−30	−32	−14	4.71
			L	−30	−38	−6	4.14
	Lateral occipital cortex	445	L	−42	−68	42	4.03
	Angular gyrus		L	−44	−50	22	3.01
	Lateral occipital cortex		L	−48	−60	42	3.77
	Middle temporal gyrus	190	L	−60	−8	−22	3.96
			L	−52	−24	−8	3.86
			L	−52	−18	−14	3.51
	Precuneus	494	R	0	−48	38	3.86
			R	12	−54	24	3.73
			L	−4	−60	20	3.72
**L-DN**	Precuneus	1851	L	−12	−60	48	5.39
			L	−4	−62	62	5.20
			L	−12	−46	42	4.80
	Supramarginal gyrus	251	L	−54	−46	40	4.01
	Lateral occipital cortex		L	−44	−64	44	3.00
**R-DN**	Precuneus	3453	R	8	−78	48	6.02
			L	−12	−60	48	5.51
	Lateral occipital cortex		R	18	−72	48	5.27
	Supramarginal gyrus	323	L	−46	−46	44	4.46
	Lateral occipital cortex		L	−44	−62	44	4.39
	Angular gyrus		L	−50	−54	46	4.09

Peak Z-score of the peak voxels showing greatest statistical differences in a cluster are reported in MNI coordinates (x, y, and z). Results are considered significant after correction for multiple comparisons (FWE-corrected *p* < 0.05). NoV, number of voxels; L, left; R, right; MNI, Montreal Neurological Institute; DN, dentate nucleus.

## Data Availability

The data presented in this study are available on request from the corresponding author.

## Supplementary Materials

The following supporting information can be downloaded at: https://www.mdpi.com/article/10.3390/biomedicines10112901/s1.
