# Supplementary material for: The Cerebellum Is a Key Structure in the Neural Network for Mentalizing: An MRI Study in the Behavioral Variant of Frontotemporal Dementia
Source: Biomedicines. 2022 Nov 11;10(11):2901. doi: 10.3390/biomedicines10112901 (PMC9687564; doi:10.3390/biomedicines10112901)
Supplement: Supplementary file 1 [file biomedicines-10-02901-s001.zip › biomedicines-1954041-supplementary.pdf]

Neuropsychological assessment of FTD patients

In order to assess the general cognitive profile, each patient was administered an extensive neuropsychological examination, including tasks of visual and verbal memory (Rey's Auditory Verbal Learning Test, RAVLT) including subtests of immediate and delayed recall and forced-choice recognition (Carlesimo et al., 1996), Rey-Osterrieth Complex Figure recall (Caffarra et al., 2002a), short-term verbal (digit span) and visuospatial memory (Corsi block-tapping task) (Monaco et al., 2013); phonological (F, A, S) and semantic (birds, furniture) verbal fluency (Quaranta et al., 2016); copy of Rey-Osterrieth complex figure (Caffarra et al., 2002a) executive functions (Stroop's test, Caffarra et al., 2002b); Modified Wisconsin Card Sorting Test (MWCST, Caffarra et al., 2004); Trail Making Test (Giovagnoli et al., 1996); visual attention (Multiple Features Targets Cancellation, MFTC) (Marra et al., 2012); abstract reasoning (Raven's Coloured Progressive Matrices—PM'47, Carlesimo et al., 1996); copy of pictures with and without landmarks (Carlesimo et al., 1996).

Neuropsychological results

As showed in Table 1, bvFTD patients presented slight cognitive deficits. In particular pathological scores were obtained in verbal (RAVLT immediate and delayed recall as well as forced-choice recognition accuracy), and visual memory (Rey-Osterrieth figure copy), visuospatial abilities (Rey-Osterrieth figure recall and Copy of figures with landmarks) and executive functions (Stroop’s test: interference time and WCST perseverative errors).

References

Caffarra, P., Vezzadini, G., Dieci, F., Zonato, F., Venneri, A., 2002a. Rey-Osterrieth complex figure: normative values in an Italian population sample. Neurol Sci. 22,443–447.

Caffarra, P., Vezzadini, G., Dieci, F., Zonato, F., Venneri, A., 2002b. Una versione abbreviata del test di Stroop: dati normative nella popolazione italiana. Nuova Riv Neurol. 12,111–115.

Caffarra, P., Vezzadini, G., Dieci, F., Zonato, F., Venneri, A., 2004. Modified Card Sorting Test: normative data. J Clin Exp Neuropsychol. 26(2),246-50.

Carlesimo, G.A., Caltagirone, C., Gainotti, G., 1996. The Mental Deterioration Battery: normative data, diagnostic reliability and qualitative analyses of cognitive impairment. The Group for the Standardization of the Mental Deterioration Battery. Eur Neurol. 36(6),378-84.

Giovagnoli, A.R., Del Pesce, M., Mascheroni, S., Simoncelli, M., Laiacona, M., Capitani, E.,1996. Trail making test: normative values from 287 normal adult controls. Ital J Neurol Sci.17(4),305-9.

Marra, C., Gainotti, G., Scaricamazza, E., Piccininni, C., Ferraccioli, M., Quaranta, D.,2013. The Multiple Features Target Cancellation (MFTC): an attentional visual conjunction search test. Normative values for the Italian population. Neurol Sci. 34(2),173-80.

Monaco, M., Costa, A., Caltagirone, C., Carlesimo , G. A., 2013. Forward and backward span for verbal and visuo-spatial data: standardization and normative data from an Italian adult population. Neurol. Sci.34, 749-754

Quaranta, D., Caprara, A., Piccininni. C., Vita, M.G., Gainotti, G., Marra, C., 2016. Standardization, Clinical Validation, and Typicality Norms of a New Test Assessing Semantic Verbal Fluency. Arch Clin Neuropsychol. 31(5),434-45
